# ‘You feel part of something bigger’: Stakeholders’ experiences of a long-term community–academic participatory research partnership

**DOI:** 10.1177/13623613251348485

**Published:** 2025-06-19

**Authors:** Elizabeth Pellicano, Catherine A Bent, Teresa Iacono, Kristy Capes, Shannon Upson, Kristelle Hudry

**Affiliations:** 1 University College London, UK; 2 La Trobe University, Australia

**Keywords:** community–academic partnership, community engagement, early intervention, early years, participatory research, service evaluation

## Abstract

Many community–academic partnerships are established to conduct meaningful research and practice. Yet, little is written about their sustainability and what does exist suggests that effective partnerships are hard to sustain. In this study, we sought insights into what might support successful, enduring community–academic partnerships focused on early childhood autism service provision. We conducted semi-structured interviews and focus groups with 30 staff connected to a university-based early childhood service in education, allied health, management, and research roles, who had been involved in a decade-long community–academic partnership. We analysed the data using reflexive thematic analysis adopting an inductive approach. We identified three key themes. Staff shared strong values and commitments towards inclusive practice and evidence-based practice, which were embedded in the ethos of the partnership (Theme 1). They reported tangible, mutual benefits from working in partnership, with gains in learning and confidence supporting autistic children and families (Theme 2). Interviewees also highlighted the importance of strong equitable relationships, open communication, and fair processes, even if these were not always straightforward to achieve (Theme 3). These insights illustrate the value of ongoing monitoring of community–academic partnerships seeking to inform autism research programmes, policies and practices, while also building sustained community capacity.

**Lay Abstract:**

When academic researchers work in partnership with community members, the research that gets done is usually more meaningful to people’s everyday lives. But these ‘community–academic partnerships’ can be difficult to set up, and even more difficult to keep going. In this project, we wanted to know what factors help to support the success of long-term community–academic partnerships, specifically for early childhood autism services. We spoke in depth to 30 staff connected to a university-based early childhood service, including early childhood educators, allied health professionals (psychologists, speech pathologists, occupational therapists), people managing the service and researchers. All had been involved in a community–research partnership that had been going on for a decade. Two researchers independent of the service led the interviews and analysis, looking for patterns in participants’ responses. We identified three main ideas or ‘themes’. Staff spoke of their strong values and commitments towards inclusive practice and evidence-based practice, which were shared among those within the partnership (Theme 1). They felt they had learned a lot from being involved in the partnership and had gained confidence supporting autistic children and families (Theme 2). Above all, though, they spoke of how the relationships within the partnership really mattered to making it a success. They emphasised the importance of trust, good communication and fair processes – but also noted that these things were not always achievable (Theme 3). These findings help us understand how researchers and community members can work effectively together to bring lasting benefits to autism research and services, and to the community more broadly.

Research can be fundamental to improving health and well-being. Yet, the translation of discoveries into benefits for human health can be frustratingly slow (Balas & Boren, 2000; Ioannidis, 2006). This research-to-practice gap is especially stark in the field of autism, where, despite enormous investment in autism science, the life outcomes of autistic people remain severely constrained (Howlin & Magiati, 2017; Lord et al., 2022; Pellicano et al., 2022). A primary challenge facing autism science is to reduce this gap, ensuring that scientific discovery translates into meaningful improvements to the lives of autistic people.

Tailoring interventions beyond the settings in which they originated, however, can be difficult (Brookman-Frazee et al., 2010; Stahmer et al., 2019). Community-based settings – where people live, work and play – are much less controlled than conventional research settings, and a range of contextual factors can influence how successfully an intervention or programme is transported into such settings (Mazzucca et al., 2021). One way to accelerate translation is to have greater community involvement in the processes that shape both research and its clinical implementation (Collins et al., 2018; N. J. Williams & Beidas, 2019). In community-based participatory research (Israel et al., 2006; Jones & Wells, 2007), community partners work alongside academics, contributing their ‘practical wisdom’ to make research more directly relevant to people’s everyday lives (Lloyd & White, 2011). This can be particularly important with communities that have historically been marginalised by research (Benoit et al., 2005; Bonevski et al., 2014).

While building and sustaining collaborative partnerships is essential to closing the research-to-practice gap (Drahota et al., 2016), it is complex. Such partnerships require significant time and resources, cultural openness and inclusivity, clarity around expectations and concerted efforts to reduce power differentials (Nicolaidis et al., 2011; Pellecchia et al., 2018). When done well, community–academic partnerships can have enormous benefit – to the community members involved, to academic researchers and to the research itself (Brett et al., 2014; Jagosh et al., 2012, 2015). When done less well, however, partnerships can be ineffective and burdensome. This is especially the case when involvement is tokenistic in nature (Staley, 2009), which can even cause harm (Brett et al., 2014).

The few existing studies that focus on understanding the critical factors that contribute to the success of long-standing partnerships suggest that partnership members often emphasise the *outcomes* of partnerships, including measurable research outcomes and tangible community and health benefits, as key indicators of success (e.g. Israel et al., 2020; Lindquist-Grantz & Vaughn, 2016). Success goes far beyond long-term outcomes alone, however (Brookman-Frazee et al., 2012; Jagosh et al., 2015; Khodyakov et al., 2011; Wallerstein et al., 2008). Conceptual models also highlight the importance of *intermediate* outcomes of partnership effectiveness, which are shaped by the individual characteristics of partners (e.g. degree of commitment, willingness to share power, active engagement), relational dynamics between partners (e.g. trust, mutual respect, attention to power imbalances), partnership characteristics (e.g. shared and trustworthy leadership, ability to adapt/respond and effective communication strategies) and partnership processes (e.g. effective conflict resolution, communication and decision-making; Brush et al., 2020). Building and maintaining trust between partners is held to be at the crux of these models (Brookman-Frazee et al., 2012; Jagosh et al., 2015; Khodyakov et al., 2011). Indeed, a systematic review on the key characteristics of community–academic partnerships showed that the most frequently cited facilitating influences on the collaborative process were trust, respect and good relationships (Drahota et al., 2016).

These general characteristics also appear to be critical to developing and sustaining partnerships within the field of autism research. Despite an initially slow start (Jivraj et al., 2014), there is now a growing movement towards researchers working with members of the autistic (composed of autistic people) and autism communities (allies and those with an interest in autism research/practice, including parents/caregivers of autistic children, professionals and researchers) as collaborators in research (see Tan et al., 2025, for review). Excellent examples include long-term partnerships with autistic people, such as AASPIRE (Nicolaidis et al., 2011), and with the broader autism community (practitioners, funding agency representatives, researchers and family members), such as in the BRIDGE Collaborative (Brookman-Frazee et al., 2012). The few studies that have reflected upon lessons learnt from such partnerships have suggested that effective partnerships depend on strong relationships – that is, those in which there is mutual trust between partners, respect for each other’s expertise and ways of being, and an openness to learning from one another (Fletcher-Watson et al., 2019; Jose et al., 2020; Le Cunff et al., 2023; Maye et al., 2022; Nicolaidis et al., 2019; Stark et al., 2021).

Brookman-Frazee and colleagues (2012, 2016) formalised this knowledge into a model based on their own experiences of developing the BRIDGE Collaborative – partners who came together to enhance community-based care for autistic young children. Akin to work outside autism (Jagosh et al., 2015; Khodyakov et al., 2011; Wallerstein et al., 2008), their model highlights the importance of successfully navigating interpersonal and organisational processes, such as effective communication, task delegation and management of time/logistical issues (see Brookman-Frazee et al., 2016).

There are, however, remarkably few studies both within and beyond the field of autism research and practice that have focused on partnerships that were not sustainable in the long term (see Lindquist-Grantz & Vaughn, 2016, for an exception). Here, we sought to build on this work and to contribute to frameworks that conceptualise community–academic partnership success (Israel et al., 2020). Specifically, we sought to understand which factors contributed to the workings of a decade-long community–academic partnership focused on community-based early autism intervention – a partnership that was initially successful and enduring, but where challenges had evidently arisen over time. To address this issue, we used in-depth interviews and focus groups to elicit the views and experiences of staff working across a range of professional, management and research roles to understand the factors that helped and hindered the sustainability of this partnership.

## Methods

### Context

The community–academic partnership in question began in 2010, focused on a university-affiliated community children’s centre, offering a specific, manualised Naturalistic Developmental Behavioural Intervention (NDBI), Early Start Denver Model (ESDM; Vivanti et al., 2019), for preschool-aged autistic children in the context of long-day-care services in distinct but co-located, settings: (1) an ‘inclusive’ programme, in which one to three autistic children were enrolled alongside primarily non-autistic peers in regular childcare; and (2) an ‘autism-specific’ programme, in which playrooms were attended exclusively by autistic children. The centre housed a team of specialist educators and allied health professionals (i.e. psychology, speech pathology, occupational therapy), including staff certified as ESDM trainers (see also Bent et al., 2023). The service was funded from 2010 to 2018 by the Australian Government, where the intervention costs were initially covered by the government grant. This changed in 2017, with the launch of the National Disability Insurance Scheme (NDIS^1^), when parents could apply for NDIS funding towards intervention costs, while self-funding childcare fees as usual.

#### Service provision context

Across the decade-long period, up to 10 autistic children attended each of two autism-specific playrooms on any given day. Prior to 2015, children could attend up to 5 days per week (for 5–8 h per day), whereas from 2015, attendance was mostly capped at 3 days per week for 1 year, increasing the number of families able to access the service. In 2015, the centre began to offer places for an inclusive programme, accommodating up to three autistic children across several inclusive playrooms, alongside 10 to 12 non-autistic children, as part of a randomised controlled trial (RCT) initiated by the community–academic partnership (Vivanti et al., 2019). The inclusive programme adopted staff-to-child ratios in line with government regulations (1:4 in <3-year-olds; 1:11 in >3-year-olds). The autism-specific programme had a higher staff-to-child ratio (4:10), which fluctuated across the day (e.g. 1:2 for small group experiences). From 2015, each setting had one certified ESDM therapist trained to support other early childhood educators who had attended training workshops and who received ongoing coaching from certified staff and the on-site allied health team.

The same NDBI programme, group-based ESDM (G-ESDM), was implemented across all years of the service operations, and across autism-specific and inclusive programmes. Each autistic child/family in each programme (autism-specific, inclusive) was allocated a primary and secondary keyworker – an allied health professional or certified educator – responsible for liaising with families, developing and reviewing children’s goals, and modifying their individual programme. Children’s individual learning goals in communication, play, social engagement and cognition were targeted throughout daily routines and group activities. These goals were developed collaboratively with parents following an initial child assessment. Staff recorded data daily using an electronic device to allow prospective tracking of, and individualised support towards, children’s goals. Keyworkers reviewed children’s data weekly, making programme adjustments as required.

The training team for the research evaluation included two ESDM trainers and an ESDM certified and experienced educator who specifically supported coaching into the inclusive setting. Allied health and lead educators (lead and lead support) in the autism-specific setting were trained and certified in the model, as per the certification process, and were also trained to deliver the therapy in a group setting, according to G-ESDM protocols.

Educators in the inclusive setting and the other educators in the autism-specific setting (aside from the lead and lead support) were trained in G-ESDM, but not certified. The training included a 3-day ESDM paraprofessional workshop, including tailored coaching with autistic children in the autism-specific/inclusive programmes to embed children’s individual learning objectives within daily routines and curriculum experiences. Educators in inclusive settings also received ‘top-up’ training via in-person workshops (one to two times annually) in response to their needs (identified by surveys, interviews and fidelity scores). They also received ongoing informal coaching and support from the children’s key worker.

All staff were regularly assessed by the training team against a fidelity tool. These assessments occurred for all staff trained in the model, including certified, non-certified, educators and allied health. Feedback was provided and coaching sessions were provided to staff identified as not at fidelity.

Finally, as with many early childcare settings (see Community Early Learning Australia, 2021), the service was subject to considerable staff turnover and there were also multiple changes in key staffing positions, including new (clinical manager and researcher) and promoted staff (one trainer became centre manager and one key worker became assistant manager). The training team reduced from two people to one person for a full year of the project, reducing capacity to provide time and support to the education team. During the current research period, the service was in the early stages of change management.

#### Research and evaluation context

Families attending the service were routinely invited to participate in research activities. This included research for a government-mandated core service evaluation broadly aimed at evaluating the feasible and appropriate delivery of the service to meet child and family needs. Initial government funding included provision for one full-time post-doctoral researcher, employed by the university and co-located on-site at the children’s centre, and required the collection of direct examiner-administered and parent-report assessments at children’s intake, and repeated at yearly intervals (i.e. approximate end of each school calendar year) and/or ahead of their exit from the service.

The staff researcher, together with academic and community partners, also devised and secured additional funding for other, hypothesis-driven research. This included submissions in response to Commonwealth Government targeted competitive funding calls for aligned projects, expanding on the mandated evaluation – to test more specific questions about the intervention approach (i.e. the RCT of autism-specific vs inclusive programme delivery) and inform understanding of development in early childhood autism and related conditions (i.e. other prospective observational work). The acquisition of subsequent project funding by the community–academic partnership facilitated the employment of additional research support staff.

### Participants

For this study, we invited the following stakeholders who had been involved in programmes that support autistic children at the centre between 2010 and 2019, including those who were no longer employed/involved in the service where possible:^2^ (1) educators working directly with children in inclusive or autism-specific settings (n = 17), and (2) members of the training and supervision team who providing, coaching and supervision to educators (n = 8); (3) managerial staff (n = 5) and (4) researchers involved in the conduct of research as part of the community–academic partnership (n = 8). Given the time that had passed, not everyone was contactable. Of the 38 participants to whom we reached out, 30 agreed to participate, including 25 (autism) community partners (educational or clinical professionals and managers) and five academic partners (formally trained researchers). Almost all were women (97%). Among the 25 community partners, there were 15 early childhood educators (minimum qualification Certificate III in Early Childhood Education and Care^3^); 6 clinical staff with psychology, speech pathology or occupational therapy backgrounds who formed the allied health/training team; and 4 managerial staff with clinical or educational qualifications. These staff were at various career stages and reported employment at the centre spanning a wide range of years (M = 4.78 years, SD = 2.95; range = 1–10). Academic partners ranged from doctoral students through post-doctoral researchers, and their employment within the partnership ranged from being research assistants through to tenured faculty collaborators. On average, these staff were affiliated with the children’s centre for 5.8 years (SD = 3.56; range = 2–10). Many participants self-identified as culturally/linguistically diverse, including one-third (mostly educators) who reported Central or South-East Asian ethnicity.

### Procedure

Ethical approval was granted by La Trobe University Human Ethics Committee (Reference Number: HEC18514). Participants provided informed, written consent for their participation. We had planned to conduct focus groups with all stakeholders, with each group kept exclusive (i.e. educators only, trainers/managers only, researchers only) to minimise the impact of potential unequal relationships on individuals’ responses. Some participants, however, preferred individual interviews. We therefore conducted four focus groups with educators (M = 52.5 min duration; range = 50–56), four focus groups and three individual interviews with allied health professionals and managers (M = 50.4 min; range = 39–65), and five individual interviews with researchers (M = 41.7 min; range = 23–51).

Focus groups (with two to five staff) were conducted in person at the centre in 2019, while individual interviews were conducted online. They were facilitated by one or two researchers (E.P. and T.I.), who were neither affiliated with the centre nor well known to participants. Facilitating researchers posed a series of open-ended questions about their respective roles, their views and experiences of supporting autistic children (and/or staff) in specialised and mainstream inclusive settings, and their knowledge of, or involvement in, the community–academic partnership (see Supplementary materials). The questions were reworded to suit their different roles. For example, educators were asked, ‘What were your goals and aspirations for the autistic children with whom you worked, and how did you go about supporting the children to achieve those goals?’ while researchers were asked ‘What were your experiences of working with educators and allied health professionals to support children on the autism spectrum?’ Participants were sent the primary questions in advance. All interviews/focus groups were recorded with participants’ prior permission and transcribed verbatim by a transcription service.

### Data analysis

We followed Braun and Clarke’s (2019) method for reflexive thematic analysis, within a critical realist framework (Willig, 2013), which acknowledges the broader social context in which individual meanings are constructed and mobilised. In so doing, we used an inductive (bottom-up) approach to identify patterned meanings within the dataset.

Analysis began during the interview phase, where the lead facilitators wrote reflexive notes, and met regularly to debrief and discuss patterns in participants’ responses. Following transcription, E.P. immersed herself in the data, reading all transcripts twice, taking reflexive notes on striking and recurring observations and applying codes to each transcript (managed in NVivo, version 12). Codes were clustered together to identify candidate themes and subthemes. E.P. then generated a draft thematic map depicting the themes and subthemes, which was shared with T.I. and other members of the team and then discussed and reviewed multiple times with the broader team, focusing on semantic features of the data. Themes and subthemes were identified through systematic engagement with the data and an active, deeply reflexive approach to analysis, influenced by the researchers’ own aims, positionalities and interpretation.

### Positionality statement

Our analysis was informed by a diverse range of perspectives, each shaped by our distinctive professional and academic backgrounds, including expertise in educational psychology, psychology and public health, speech-language pathology and early childhood education. The authors came together for this project because they felt it important to draw key lessons from this specific community–academic partnership to help guide future partnerships and autism practice research. Two professionals (K.C. and S.U.) from education and allied health backgrounds involved in service delivery and management had contributed to the community–academic partnership since its inception; two researchers (K.H. and C.B.) had worked on research conducted within the partnership but had not been involved from the outset, and two researchers (E.P. and T.I.) were not affiliated with the partnership at all, or with the children’s centre. These latter two researchers conducted all interviews and focus groups and were primarily involved in data analysis.

### Community involvement

This study drew upon a long-standing community–academic participatory research partnership, involving people in research, professional and management roles. All authors – professionals and researchers – worked together from the outset of this project to (1) secure funding for the project, which included reimbursement to the service for educator time while they participated in the research during working hours; (2) develop the ethics application; (3) design the interview and focus group schedules; (4) input into the analysis and interpretation of the findings; and (5) contribute to the write-up of the manuscript (all are named authors). Professionals and researchers involved in the partnership also shared their own experiences as participants in the research reported here. Notably, no autistic people or parents of autistic children were involved in this design of this research study – or in the design of the partnership itself.

## Results

**Figure 1.**
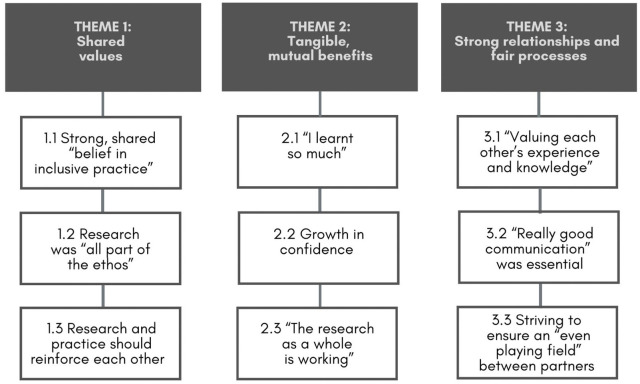
Interviewees’ views and experiences of participating in a community–academic partnership: themes and subthemes.

We identified three themes – key factors – that were understood to have either helped or eventually hindered the longevity of the partnership. Figure 1 shows the themes and associated subthemes, which are numbered below and presented in bold and italics, respectively. Illustrative quotes are also provided, attributed via informant type (AH: allied health; EDU: educator; MGMT: management; RES: researcher).

### Theme 1: shared values

Interviewees repeatedly spoke of common goals and the importance of a shared ‘value system around child outcomes and family outcomes’ (MGMT). At its core, they reported a *strong, shared ‘belief in inclusive practice’ (subtheme 1.1)* – that is, ‘having [autistic and non-autistic] children interact with each other and learn from each other’ (AH) – and ‘could see how inclusion was good for everyone’ (RES). One researcher recounted how this shared ‘commitment and theoretical orientation toward fostering inclusion for autistic children’ (RES) underpinned the establishment of the partnership and that this ‘investment in inclusion’ persisted. Professionals described wanting autistic children to have ‘the same things that we want for every other kid – fantastic friendships and great lives and great input into the community’ (MGMT), to ‘maximise their capabilities and develop their skills to integrate into society’ (MGMT). They also spoke of being ‘super committed’ (AH) to ensuring that children are able ‘to express themselves’ and that ‘they are part of what happens’ (AH).

Regarding individual autistic children, interviewees spoke of focusing on ‘encouraging independence’ (EDU), as well as ‘making friends and social connections’ (EDU): ‘seeing that is always lovely, whether it’s participating in a game of chasey or playing playdoh – just being in that environment where they’re interacting’ (EDU). They also sought to expand the children’s ‘current interests and open them up’ (AH): ‘I really love it how [child] didn’t even know what toys were and now he actually opens cupboards and looks . . . It’s showing them another world outside of the world they know’ (EDU).

According to interviewees, this strong belief in giving children ‘the best opportunity’ (AH) was underpinned by *research being ‘all part of the ethos’ (subtheme 1.2)*. They explained how working within a university-based children’s centre meant that ‘research underpins everything’ (RES). While some felt it could ‘take a while for people to get used to [the research]’ (RES), our community partner interviewees appeared to have embraced it, describing how ‘being able to combine research and practice was one of the biggest things that made me want to work there’ (AH). This sentiment was reportedly shared by parents: ‘a lot of [families] would say that the research program was part of why they wanted to be there’ (MGMT). These ‘unique conditions’ (RES) also gave the staff purpose: ‘it’s different because I feel like it’s not just with these [autistic] kids. I’m doing something bigger’ (EDU). Another agreed: ‘you can tell your job’s important, and you want to do it better’ (AH). They also spoke of how it gave them ‘a bit of a buzz’ (MGMT) being embedded in a research culture and that ‘always having these sorts of conversations’ (AH) gives you ‘a better understanding about what we are doing and also [about] the children themselves’ (EDU). Consequently, they felt ‘really proud to be part of this centre and of the research’ (EDU).

Interviewees also showed a strong belief that *research and practice should reinforce each other (subtheme 1.3)*. Many reported their ‘huge commitment to ESDM’ (MGMT) in part because they felt it ‘is very flexible, adaptive and functional-based’ (AH) and ‘provides a lot of support to young children and puts families at the heart’ (MGMT) and in part because they believed it ‘had a strong evidence base’ (RES). While they acknowledged that ESDM was not ‘going to suit all children’, they nevertheless ‘felt quite strongly’ that both research and practice would show ‘it was going to improve education’ (MGMT).

Despite their strong belief in the need to combine insights from research and practice, interviewees were aware that this was not always straightforward, especially given the intensity of work required by the intervention, which ‘was a big demand’ (MGMT): ‘it’s a fantastic model, but it’s pretty resource-heavy to train and then maintain skill sets’ (MGMT). Clinical professionals were felt to be ‘very much on board because they . . . had a better idea of what to expect in their roles’ (AH). But the same commitment was not felt by educators, particularly within the inclusive programme. While interviewees reported ‘some of [the educators] thrived on it and enjoyed the change and discovered a real flare for ESDM’ (MGMT), ‘a lot of them just struggled to find the time to do ESDM on top of their jobs’ (AH) – a sentiment with which the educators agreed. One reported ‘we feel it’s a bit challenging’ (EDU) and even said, ‘I’m exhausted’ (EDU). The data collection techniques and ‘having all these extra data to take a look was very stressful on us educators’ (EDU). They felt they ‘were spending a lot of time on that kind of stuff instead of focusing on the group with the child’ (EDU), to the extent that some felt they ‘neglect the other [non-autistic] children in the room’ (EDU). They also questioned whether implementation into the mainstream childcare context had been fully considered: ‘sometimes people in research are disconnected from the people that they’re working with’ (AH). One educator elaborated:

. . . you have a child in the room toilet training, or you have one having a tantrum over there, or one getting dropped off by the mum, so they deserve your 100% attention. That takes three educators away. So as much as the program sounds amazing on paper, once you go into the rooms and you see it hands-on every day, everything’s different. (EDU)

Perhaps because of these ‘practicalities and the cost of working in that way, the centre went in a different direction with the [program]’ (RES) once the RCT had been completed. This same interviewee explained that ‘the decision was that it wasn’t necessarily feasible to continue in the model as it was, because it wasn’t providing a cost-effective solution’ (RES). The changes meant that ‘therapy assistants embed the ESDM within the mainstream childcare, so now the educators no longer have to take data and work on the child’s specific ESDM learning objectives’ (AH).

### Theme 2: tangible, mutual benefits

Further to the perceived importance of shared beliefs and values, interviewees also spoke of the role that tangible benefits played in helping to secure the partnership over time. This began with the benefits of publication and grant funding. Interviewees recounted how ‘in designing the research, we collectively as a team decided . . . that we want to be able to publish something of a really high standard’ (AH) and community partners were proud of ‘a paper already published’ (MGMT) on which some were ‘named authors, out of recognition for the work we put in’ (MGMT). Yet, it was the learning that all parties had done throughout the partnership that resonated most. In one educator’s words, *‘I learnt so much’ (subtheme 2.1)*. One researcher explained that, from the outset, they perceived ‘staff development opportunities’ to be intrinsic to the partnership: ‘it’s not that researchers are coming in, imposing something . . . it needs more commitment and taking them off the floor to train them, but they’re going to be more skilled going forward’ (RES). Interviewees agreed that they ‘learnt so much in such a short period of time’ (EDU), and that they felt ‘challenged, that there’s different ways to do different things’ (EDU). Some educators reflected on how getting feedback and being able to reflect on their performance was ‘really, really important . . . in that way, we learn more and get better at it’ (EDU).

Educators in the inclusive programme felt their learning generalised to how they taught and supported non-autistic children, too: ‘lots of the strategies we are using here, it also works well on mainstream children’ (EDU). Others agreed they had ‘added to their skill set’ (MGMT) and given them a ‘sense of closer observation, setting objectives for individual children’ (AH): ‘it’s more child-centred, so you’ve got all these teaching strategies you can use with the children’ (EDU).

The importance the interviewees placed on learning also went beyond the implementation of the programme’s specific tools to a more general sense of self-efficacy. They described *growing in confidence (subtheme 2.2)* through the work. The training and ‘informal learning opportunities within the team’ (MGMT) made them feel they could ‘sort stuff out by ourselves’ (EDU) rather than needing to call on others’ expertise. Interviewees also felt that the community–academic partnership gave them a unique chance to feel ‘empowered and involved’ (RES) and gain a ‘better understanding’ (EDU), like ‘‘wow, how does that affect Child A that I deal with every day?’ ‘What does that mean for my teaching and my team?’’ (MGMT). It also appeared to instil a sense of ‘openness and wanting to do things better’ (AH).

Interviewees also stressed the real impact of their work on the lives of children and families: ‘I think we have research that is more meaningful and more relevant to families and to children themselves, and there’s just potential to have more impact in that way’ (RES). In short, they felt that *‘the research as a whole is working’ (subtheme 2.3)* – which they found so ‘rewarding’ (EDU). Educators reported how children ‘are very happy’ (EDU) and they were ‘calm and interacting, engaging with activities’ (EDU). They also reported that autistic children’s learning generalised across settings: ‘we’ve got lots of family feedback of things changing at home . . . which is amazing’ (EDU).

### Theme 3: strong relationships and fair processes

While interviewees felt shared beliefs and mutual benefits were necessary for ensuring ‘buy-in from everyone who’s involved in the process along the way’ (MGMT), they also acknowledged they were not sufficient for *sustaining* the community–academic partnership over time. Interviewees also emphasised the importance of building ‘deep trust’ (RES) and ‘shared understanding and respect among people’ (RES) both within the partnership and with ‘people who are championing the research outside’ (AH).

Central to these relationships was *‘valuing each other’s experience and knowledge’ (subtheme 3.1)*. Researchers described that, from the outset, they were ‘often in the playrooms doing things with [educators] and trying as much as possible to listen to what they had to say’ (RES). Others reported feeling inspired by how ‘we bounce off each other’ (EDU). There was also much acknowledgement that having ‘lots of ideas and lots of people, who contribute different ideas’ (EDU) was a ‘real opportunity . . . – you can explore those things that people notice . . . particularly the educators, who’d have great ideas about different patterns they’d noticed or things they’d like to know more about’ (MGMT). Valuing one another’s ‘training and knowledge about how to deal with all kinds of things in the room’ also made educators feel ‘supported’ (EDU): ‘there is always people on standby that we can call and ask questions or even come in to show us how it’s done’ (EDU). This meant that everyone worked collaboratively: ‘it’s not just sort of like, ok, so that’s your role and that’s my role. We all want to work together’ (EDU). Educators also described how researchers listened to, and acted on, their knowledge:

That’s been a real strength of us having quite a close relationship with the research team – we’ve been able to feed back family-specific information, whether that’s receiving feedback from a family about them feel[ing] quite anxious about some reports they’d been given and us feeding that back to the team . . . and in turn, [the researchers] changed the processes they had in place. (EDU)

The intervention fidelity checks were another key example of the importance of listening and feeling heard, and often revealed difficulties in the partnership. Interviewees explained how the fidelity checks ‘were probably the biggest stress for the teams’ (MGMT): ‘they scare the shit out of the [educators], to put it bluntly’ (AH). One interviewee explained, ‘in the ESDM, it’s accepted you’ll be assessed – filmed, watched, rated and handed a score . . . but some staff would actually call in sick on the days of their fidelity check, because they felt so anxious’ (MGMT). Educators, too, described themselves as ‘extremely anxious’ (EDU) about the fidelity checks: ‘it just feels like a test’ (EDU). It had become clear over time, however, that:

[rather than] just assuming and imposing it [fidelity checks] on staff, we ask them, ‘well, do you want to be filmed?’ . . . so we just became increasingly sensitive and responsive – getting suggestions from them along the way was helpful to tweak the process and individualise it. (MGMT)

Not all educators felt that their expertise was properly valued and they identified that as a significant challenge to the partnership over time. Some felt there was a lack of recognition of educators’ everyday experiences – ‘it’s a specific way you need to interact with children in early childhood’ (EDU) – and how integral that was to the project:

We had an incident where a child couldn’t ingest a hard pear. The therapy assistant couldn’t get the pear out of his mouth so, because I have the confidence and I’ve worked in this industry for a long time, I put my glove on and I got that pear out of his mouth. That added confidence of the therapy assistants is needed around the children. (EDU)

Developing this idea, interviewees emphasised how *‘really good communication’ was essential (subtheme 3.2)* to sustaining community–academic partnerships. They spoke of the need for ‘strong communication’ (AH), including ‘more regular check-ins or information sharing’ (AH) and ‘opportunity for open dialogue’ (AH). One interviewee explained how ‘sometimes there were whispers about different things happening – then things get tricky. So, that communication helps with transparency’ (AH).

Almost all interviewees noted the strain placed by a ‘breakdown of communication’ (MGMT) as the partnership progressed. In part, this was felt to be ‘because people just don’t have the time’ (RES): ‘it’s something we have to almost tell ourselves. Make sure we’re sharing that information because, when you’re very busy, it doesn’t happen’ (AH). It also depended on the nature of people’s roles in the partnership. One interviewee noted ‘the fact that the research team are based here, and we know them on a one-to-one basis both personally and professionally, means we’re always having those sorts of conversations’ (AH). Others noted the lack of communication with educators specifically: ‘it’s almost like a triangle, where the education team and us [allied health team] have interaction and we have interaction with researchers. But the educators and researchers don’t have any input’ (AH). Educators agreed: ‘we don’t know [which researcher] is coming in and we don’t know [which child] is going [out with them]’ (EDU). Interviewees also noted the difficulties posed when this lack of communication extended to families, especially regarding research assessments:

I don’t think it’s always clear to the family what [the research] is and what they’re to expect . . . families often don’t know what they’re doing or what the purpose of this appointment with the research team is until they’re in that appointment. (AH)

Interviewees felt that genuinely shared decision making within the community–academic partnership was integral to its potential to last, with many stressing the importance of *striving to ensure an ‘even playing field’ between partners (subtheme 3.3)*. They described how the partnership ‘started out with a great idea, a genuinely a collaborative idea’ (AH): ‘we had sit-down sessions with educators from both settings involved, there was a lot of buy-in, there was a lot of opportunity to have a voice in where we were headed and a voice in perceived or predicted challenges’ (MGMT). Another interviewee explained,

that brainstorming that we had five years ago or whenever it was, that was really great. It was like, what do you want to know for the work that you do? What do families tell you they want to know? (AH)

Those involved in the initial discussions were perceived to be ‘so collaborative’ (MGMT): ‘I think the whole team – the educators and clinicians – always got a bit of a buzz out of being involved in those meetings’ (MGMT). One interviewee recounted,

What really stood out to me – and something that I often reflect on still – is the importance of that meeting and how successful it was in getting the buy-in for – from the different people in the room. Even though [the RCT] was a difficult thing to do and there were a lot of obstacles we had to overcome to do [it], the fact that everyone in the room had committed to that and knew what they were working towards and had a say in that decision was really important. (AH)

During the meetings, one interviewee explained that the structure was such that ‘everyone had a chance to share what was particularly relevant for them, so everyone got to have a say’ (RES). But this same interviewee lamented that, as time went on, the meetings ‘stopped happening and we haven’t been involved again, which takes away that avenue to be able to share that information’ (RES). Others went so far as to say that ‘it had started to fall apart’ (MGMT), that ‘the relationships feel like [they were] deteriorating’ (RES).

Interviewees identified several ways in which it was hard to maintain this even playing field. One was that ‘there was a lot of change’ (RES), where key people ‘involved in that initial meeting simply didn’t stay on for the duration of the project’ (AH). One interviewee suggested developing ‘more protocols around how it is a collaborative process, which would support accountability more and setting up expectations – through a MOU [memorandum of understanding] or through agreed actions, outputs’ (MGMT) that may have helped to ensure ‘buy-in from people as the team keeps changing over time’ (MGMT).

Responses from other interviewees suggested, however, that power differentials between partners also intensified over time. This was in part because ‘mainstream educators probably weren’t as involved in the setting [up] of the project in the first place – they had no buy-in’ (MGMT); they ‘didn’t put their hand up to be part of that program, it’s been done *to* them’ (AH). Perhaps as a result, the educators were described by other interviewees as seeing ‘it more as an add-on of all of the other things they already had to do’ (AH), for which they ‘didn’t get a [salary] raise; they didn’t get any benefit’ (RES): ‘we’ve just thrown people into a program, who weren’t asking for that. I think that’s a lot of where it comes from’ (AH). One potential consequence of this lack of buy-in was the ‘quite high staff turnover’ (AH), particularly among educators in the inclusive programme, who needed to be ‘willing to work all year round in a very demanding role’ (MGMT). Interviewees also recounted how the allied health professionals had more opportunity to talk with the research team and be part of meetings, whereas the educators did not always have this opportunity: ‘They’re the ones who are full-time on the floor, but they don’t actually get a chance to see what it is they’ve actually done, how they’re actually making a difference to these children’ (AH). One educator explained further:

When I started working here, [it was] such an amazing opportunity to have this collaboration between clinical and research [staff] and have all this potential . . . I guess, at the moment, that potential feels non-existent. There’s not that collaboration, there’s not that communication, we’re just doing our day-to-day thing and assuming that, at some point, the data will be collected for something meaningful. (AH)

The negative effects of not feeling fully involved were felt deeply by educators. One interviewee explained that ‘sometimes the [educators] didn’t feel confident enough to contribute because the clinical team was god-like and their qualifications are so much greater . . . and [educators] felt uncomfortable about talking issues through’ (MGMT). Educators themselves reported feeling ‘guilty, because the room was so demanding’ (EDU); ‘I felt like I couldn’t live up to the expectations of the therapists; I felt totally judged’ (EDU). As a result, they compared themselves to those with little status: ‘we’re like the computers, we’re the data collectors’ (EDU). One educator went so far as to say: ‘I’m feeling very at the bottom’ (EDU).

One interviewee spoke of ways to improve the participatory process:

bringing people along for the ride is really important . . . making sure that throughout the year, there are check points so that people can see the work they’re doing, how it connects to the greater research project and how it impacts kids’ outcomes. (AH)

## Discussion

This study sought to understand the processes underpinning the sustainability of a decade-long community–academic partnership in the context of a community-based early autism intervention service. Overall, partnership members were initially excited about being ‘part of something bigger’. They highlighted the importance of members’ shared values, goals and vision, in both inclusive practice and research itself – and emphasised how they developed strong equitable relationships, in which they felt they could have an equal say ‘in where we were headed’. These findings corroborate Gomez et al.’s (2021) empirical work, which showed that interpersonal processes, including shared group vision and respectful and positive relationships between partners, were the most influential factors during the *formation* of a successful community–academic partnership.

Despite noting the importance of these interpersonal processes at the outset, it was often the more tangible outcomes that generated more attention. Interviewees pointed to traditional research outcomes (grant funding and publications); the more firsthand, mutual benefits that came from working together, including knowledge generation (of autism research and practice) and increased confidence in working with the young autistic – and even non-autistic – children in their care; and the positive impacts they felt their work had on children and families’ everyday lives. These tangible, concrete benefits are often identified by partnership members as determining whether a long-term partnership is successful, over and above partnership processes, which can sometimes be more difficult to ‘see’ and measure (Lindquist-Grantz & Vaughn, 2016).

Yet, other research has repeatedly shown that relationships and partnership processes – so-called intermediate outcomes – are in fact key to the sustainability of long-term community–academic partnerships (Brookman-Frazee et al., 2016; Pellecchia et al., 2018) and the ones that require close monitoring by partnership members to ensure their longevity. Consistent with this research, our interviewees also repeatedly highlighted the importance of valuing each other’s experience and knowledge, of ensuring clear and transparent communication, and of always striving for ‘an even playing field’. They also acknowledged that these intermediate outcomes were not always straightforward to achieve, particularly as the partnership progressed – so much so that they reported ‘cracks’ emerging in the partnership over time.

Conceptual models have emphasised the importance of trust building and maintenance in successful long-term community–academic partnerships (Jones & Wells, 2007; Wallerstein et al., 2008, 2018). Jagosh et al. (2015) also identified how trust can be conceived simultaneously as a key component of the *context* (or backdrop) of the partnership, a *mechanism* that drives the partnership forward as well as an *outcome* of the partnership. They further emphasised the dynamic nature of trust building, where the outcomes of the initial stages of the partnership inform – or have a ‘ripple effect’ on – the context for later stages. In this way, trust is seen as foundational to partnership synergy and thus partnership sustainability.

In this study, we saw the challenge of maintaining trusted relationships over time and the many different factors that can underpin trust – as Jagosh et al. (2015) described. Initially, many community partners described having little experience of being involved in research (context) but nevertheless brought a strong sense of trust to the process (mechanism), illustrated by their eagerness to be embedded in the university research culture and by their sense of common purpose with researchers. This should have placed the working relationships between community and academic partners on a strong footing, and positive effects on partnership synergy were seen in early stages, including the successful design and delivery of the RCT (outcome). This did not last, however. As the partnership progressed past this critical juncture (the completion of the RCT), changes to the partnership’s membership (with academic and community members moving in and out of the partnership, and even into different roles within the partnership) and to the design and delivery of the early intervention programme itself tested the degree of trust between partners. Community members felt that some key decisions were not communicated effectively and, critically, were not always made with their input. Some also felt that power imbalance increased over time. This inequity was experienced most deeply by the early childhood educators, who felt they were not listened to, despite being at the ‘coalface’, juggling many competing demands as they delivered the intervention to children. Indeed, they repeatedly highlighted the stress – and distress – they experienced as a result of the fidelity checks used to monitor and enhance the reliability and validity of the behavioural intervention. While there were some attempts to address these times of conflict (e.g. by re-thinking the fidelity process with educators), these attempts appeared to be insufficient to maintain trust and, consequently, the partnership was perceived to be negatively affected.

These characteristics – communication challenges, unequal distribution of power and limited participation – were also identified by Lindquist-Grantz and Vaughn (2016) in a partnership that was perceived not to be working, and demonstrate the importance of nurturing trust building and maintenance processes throughout the duration of community–academic partnerships, especially when partners or partners’ roles change. Importantly, interviewees noted several ways in which trust between partners could have been strengthened. They highlighted the need for effective and transparent communication processes and the implementation of formal processes, such as protocols documenting the collaborative processes and memoranda of understanding to ‘support accountability’ and agree on goals. Intentionally tending to interpersonal processes – and especially of building and maintaining trust among partnership members – is critical to the long-term success of community–academic partnerships (Lindquist-Grantz & Vaughn, 2016). Such partnerships should seek to monitor, or evaluate, partnership processes as part of their work to understand the impact of these processes on the success of the partnership (Israel et al., 2010; Schulz et al., 2003). Providing time and space for dialogue and the opportunity for partnership members to actively reflect on interpersonal processes are also critical for resolving conflict (Wallerstein et al., 2020; see also Chambers, 2018).

### Limitations

This study has several limitations. First, it was not designed to test existing conceptual models of how long-standing community–academic partnerships function effectively. We might have obtained a somewhat different pattern of findings had we more directly asked about specific partnership characteristics, processes and outcomes and how members perceived these factors as influencing the partnership’s success (Brush et al., 2020). That said, our data generally support such models, especially the need to be attentive to, and proactive about, nurturing interpersonal processes at all stages of long-term community–academic partnerships.

Second, we elicited academic and community partners’ views only at the end of the partnership, which meant that not all members involved were able to be included. To understand fully the factors that promote the success of community–academic partnerships, we need longitudinal data on members’ initial expectations and subsequent experiences.

Third, it is noteworthy that autistic people and family members were not included in this particular community–academic partnership. Including professionals alone in such partnerships is a contested issue (O. Williams et al., 2020), largely because they are more distal in their relationship to the research outcomes than lay community members, and may therefore do little to challenge power inequalities in research (Boveda & Annamma, 2023; see Tan et al., 2024, for discussion). Inclusion of those who stand to benefit most from partnerships like this one should be paramount in the development of future partnerships.

## Conclusion

Most autism research has been designed and conducted without any significant input from members of the autistic and autism communities. Encouragingly, this has begun to change. The potential benefits of community–academic partnerships are manifold but, as this work has shown, such research approaches are not always problem-free. Building and maintaining trust between partnership members – including steps to ensure effective communication and conflict resolution processes and means to address power imbalances between partners – appear to be essential to their success.

## Supplemental Material

sj-docx-1-aut-10.1177_13623613251348485 – Supplemental material for ‘You feel part of something bigger’: Stakeholders’ experiences of a long-term community–academic participatory research partnership Supplemental material, sj-docx-1-aut-10.1177_13623613251348485 for ‘You feel part of something bigger’: Stakeholders’ experiences of a long-term community–academic participatory research partnership by Elizabeth Pellicano, Catherine A Bent, Teresa Iacono, Kristy Capes, Shannon Upson and Kristelle Hudry in Autism

## Notes

1. The Australian federal government administered NDIS provides no-fault insurance cover for Australians aged <65 years who are born with or acquire a permanent and significant disability. The NDIS provides direct funding to individuals (or parents/caregivers in the case of children) to access support and services that are determined to be reasonable and necessary.

2. We did not, however, reach out to past educators for two logistical reasons. First, we did not know many of their forwarding contact details and, second, the focus groups were scheduled during work hours, held at the children’s centre (and the centre was reimbursed to cover staff time, as part of the grant funding).

3. This course is the minimum requirement to work in the early childhood education and care sector in Australia. It covers a range of core skills, including how to plan, implement and evaluate educational early childhood programmes, support healthy mealtimes for children, foster their development as well as legal and ethical requirements.
