# Supplementary material for: ‘You feel part of something bigger’: Stakeholders’ experiences of a long-term community–academic participatory research partnership
Source: Autism. 2025 Jun 19;29(11):2818–31. doi: 10.1177/13623613251348485 (PMC12531394; doi:10.1177/13623613251348485)
Supplement: sj-docx-1-aut-10.1177_13623613251348485 – Supplemental material for ‘You feel part of something bigger’: Stakeholders’ experiences of a long-term community–academic participatory research partnership [file sj-docx-1-aut-10.1177_13623613251348485.docx]

**Supplementary Table 1.** Interview schedules. Participants were asked all primary questions, which they received prior to the interview/focus group. Prompt questions helped the interviewer to gain more detailed information.

| **Participant** | **Primary questions** | **Question Prompts** |
| --- | --- | --- |
| **Community (staff) member** | Introductions  To start, we want you to talk a bit about your work in the centre. What was your role, and how long have you worked in this role and within the autism program? |  |
|  | What were your goals and aspirations for the children with whom you worked? And for individual children? | Can you tell me about the learning environment/playroom?  What was the extent and nature of your involvement with children in the autism specific and inclusive playrooms? |
|  | How do you go about supporting the children to achieve those goals? |  |
|  | In your experience, what seemed to work well – or not – for these children? | How did you know it works/ed well – or not?  What works/ed well when autistic children learn alongside non-autistic children?  What was difficult about it? |
|  | What type of supports do you think made your job easier? | Supports for the child and family?  Supports for you in your role?  Supports for other children in the class (if in an inclusive setting)? |
|  | Since its inception, working with researchers has been central to the Centre. Can you tell me a bit about that? How did you feel about that? | What was your role in the research?  Do you feel as though you had the opportunity to have a say in the research process?  What were some of the challenges? What sort of things would have made it easier?  How did aspects of the research project affect the way that you worked with the children or in your role? And your colleagues? |
|  | Part of why we’re talking with you today is to learn about what can be done better, in terms of the research-practice partnerships. In your view, what do you see as an ideal way forward [the research, the program, or how they’re intertwined]? |  |
| **Researchers** | To start, can you introduce yourself and talk a bit about you? | Could you say what your role has been in the design, development and/or implementation of the autism-specific and inclusive interventions and its research? |
|  | Part of this project was about developing collaborations between researchers, clinicians and parents in order to develop a program that can best support autistic children. | Can you begin by telling us about how the research-practice partnership came about and why? |
|  | Tell me a bit about your experiences of working with educators and allied health professionals to support children on the autism spectrum. | How much were the educators/allied health professionals/parents involved in the research component of the intervention?  Would you have liked them to be involved more, or less?  How do you think they felt about their involvement? |
|  | What factors do you think might be important to developing and maintaining these research-practice partnerships? | What has been difficult about working in this way?  What sorts of things would make it easier?  What might be some of the advantages of this approach? And any disadvantages? |
|  | Is there anything else you would like to share about the process? |  |
